# Severe mental illness and ophthalmic health: A linked administrative data study

**DOI:** 10.1371/journal.pone.0286860

**Published:** 2023-06-07

**Authors:** Finola Ferry, Michael Rosato, Gerard Leavey

**Affiliations:** 1 Bamford Centre for Mental Health and Wellbeing, Ulster University, Coleraine, Northern Ireland, United Kingdom; 2 Administrative Data Research Centre Northern Ireland (ADRC-NI), Coleraine, Northern Ireland, United Kingdom; SRM Institute of Science and Technology, INDIA

## Abstract

**Background:**

While evidence has emerged highlighting the potential benefits of the eye as a *window* to the central nervous system, research on severe mental illness (SMI) and eye health is rare.

**Aims:**

We examine the association of SMI with a range of ophthalmic health outcomes, and whether any relationship is modified by age.

**Methods:**

We used linked administrative data from general practitioner (GP), hospital and ophthalmic records to examine receipt of any Health and Social Care (HSC) eye-test; and (based on eligibility recorded for a sight test) any glaucoma, any diabetes, and any blindness among the Northern Ireland (NI) hospital population between January 2015 and November 2019 (N = 798,564).

**Results:**

When compared with non-SMI patients, those with SMI recorded a higher prevalence of having had a sight test, diabetes, and blindness. In fully adjusted logistic regression models, higher likelihood of an eye-test and diabetes (OR = 1.71: 95%CI = 1.63, 1.79 and OR = 1.29: 1.19, 1.40 respectively); and lower likelihood of glaucoma remained (OR = 0.69: 0.53, 0.90). Amongst persons with SMI there was evidence that the likelihood of having had an eye-test was lower in the older age-groups.

**Conclusion:**

Our study provides new evidence on ophthalmic health inequalities associated with SMI. While the study has immediate relevance to its NI context, we believe it is generalizable to wider UK health concerns. We emphasize the need for more research of this type, using large linkable electronic administrative databases to further our understanding of both health inequalities associated with SMI and poor eye health, and health outcomes in general.

## Introduction

It is estimated that severe mental illness (SMI), such as schizophrenia and bipolar disorder, will affect around 1% of the general population at some point in their lifetime [1]. Several studies have evidenced widespread physical health disparities, including higher rates of life-limiting chronic physical conditions, lower engagement with services and premature mortality among individuals with SMI [2–5]. Despite an established evidence base highlighting an increased risk of a range of chronic conditions such as obesity, asthma, diabetes, heart disease and stroke [3, 4], little is known about the association between SMI and ophthalmic health. Some evidence has emerged over the last decade highlighting the link between eye health and mental health, and the potential benefits of the eye as a *window* to understand central nervous system conditions [6, 7]. Liu et al found that both neurovascular diseases and glaucoma were associated with increased risks of bi-polar disorder and schizophrenia, while an earlier study also found an increased risk of anxiety and depression among individuals with glaucoma [8, 9].

Research on mental health as a *risk factor* for poor eye health is rare, particularly for SMI [10]. However, some recent studies suggest that emotional distress may be associated with the progression of ophthalmic conditions such as glaucoma [11, 12]. Shin et al highlight mechanisms that may contribute to this link, focusing on how the physiological response to stress stimulates the autonomic nervous system (ANS), which plays a vital role in the development of eye conditions [11]. However, previous studies were based on small sample sizes with few focused on SMI.

The reasons underlying the association between SMI and poor physical health are multi-factorial and include health behaviors, side-effects of medication, difficulties in accessing and engaging in treatment, as well as socio-economic determinants [13]. Given wider health disparities associated with SMI [2, 5, 13], it seems likely that people with SMI have fewer routine contacts with specialist ophthalmic services and are more likely to have advanced eye disorders when they have contact.

This study uses large routinely available administrative health-based databases from Northern Ireland (NI) to examine the association between SMI, selected ophthalmic health outcomes and socio-demographic context. It is the first administrative data study of SMI and ophthalmic health in the United Kingdom (UK). Given the relatively low prevalence of SMI in the general population, the large population base available to this study allows an in-depth examination of relatively rare health outcomes (such as SMI).

## Materials and methods

This study is part of a relatively recent Administrative Data Research (ADR) initiative to develop the use of routinely collected administrative data for research purposes. The population of interest (the *spine* of the study) is drawn initially from a database of everyone in NI registered with a General Practitioner (GP) (at the beginning of January 2013). This spine, which also contains some basic demographic information associated with each person, is (separately) electronically linked with routinely collected data on (a) hospital-based Patient Administration System (PAS) records on hospital admissions and (b) ophthalmic health outcomes, derived from Ophthalmic System (OS) data, both provided for research purposes through the Health and Social Care Northern Ireland (HSCNI) Business Services Organisation (BSO). This electronic linkage of the three administrative databases forms the data used in the analysis.

### Data

A total of 1,460,360 individuals aged fifteen years and over were registered with a GP in January 2013. These records were linked with PAS data (covering the period January 2013 to November 2019) and OS data (January 2015 to November 2019). While the hospital data includes information on dates of both admissions and discharges, this study focusses mainly on data defining primary and secondary diagnoses associated with each hospital episode. Of these 1.46 million GP patients, 798,564 also attended as hospital patients during the follow-up period. A total of 410,216 (51.4%) of these hospital patients appeared on OS data, as identified by any recorded HSC eye-test. The remaining 388,348 individuals were therefore assumed not to have used any ophthalmic services and coded as zero on all outcome variables to facilitate analysis. OS data includes information on HSC sight tests based on claims submitted to BSO for payment to the Family Practitioner Services (FPS) by primary care opticians. Given this, the figures exclude privately paid-for tests. The following groups were eligible for an HSC sight test: patients aged sixty years or over; children aged under sixteen years; students age 16–18 years and in full time education; persons receiving benefits such as income support or pension credit; adults on low incomes; patients registered blind or partially sighted; glaucoma and diabetes sufferers; people aged over forty years who are closely related of a glaucoma sufferer; and patients requiring complex lenses [14].

Ethical approval was not required for this study as it was facilitated via the Health and Social Care Northern Ireland (HSCNI) Honest Broker Service (HBS). The research related to HSCNI datasets, for which a Memorandum of Understanding is in place to permit HSCNI datasets to be used for research purposes. Research limited to secondary use of information previously collected during normal care (without an intention to use it for research at the time of collection) is generally excluded from Research Ethics Committee review, provided that the patients or service users are not identifiable to the research team in carrying out the research.

Consent was not required for this study as data was provided in anonymized format, meaning the rights of individuals are respected with adequate privacy protection. Access was provided to the de-identified data via a secure research environment and members of the research team were required to sign a Research Data Access Agreement and Disclosure Policy Agreement prior to gaining access to the data. These contracts and organizational controls ensure no individual can be re-identified during the analysis or in any of the outputs.

### Measures

#### Outcomes

Four key dichotomous outcomes (0 = no, 1 = yes) were defined based on OS data: a summary variable identified *any ophthalmic service engagement* between January 2015 and November 2019 (based on attendance at any eye-test); and three broad ophthalmic-related health outcomes derived from ophthalmic records based on the exemption category recorded on sight test claim forms—any *glaucoma*, any *diabetes*, and any *registered blindness*. Individuals not appearing on OS records (n = 388,348) were coded as zero for both HSC sight test and each of the three outcomes.

#### Explanatory variables

The presence of *any SMI* was determined from International Classification of Disease (ICD-10) codes held as part of the extensive diagnostic codes associated with the patient hospital record (PAS). SMI is defined as a dichotomous indicator (0 = no, 1 = yes) identifying individuals with ICD codes F20-F29 (schizophrenia, schizotypal and delusional disorders) or F31 (bipolar disorder) recorded as their primary or subsidiary diagnosis at any point between January 2013 and November 2019. This classification is in line with previous definitions [13, 15].

Individual characteristics available from administrative data and known to be associated with SMI and/or ophthalmic health were controlled for in the models. These include: sex (male, female); age-group (15–24, 25–34, 35–44, 45–54, 55–64, 65–74 and 75 or more years); marital status (married/separated, single, divorced, widowed and unknown); locale of residence (urban, intermediate, and rural); and deprivation decile (1 = most deprived to 10 = least deprived) [16].

#### Analysis

We examined population characteristics associated with persons aged fifteen and over (as of January 2013) and appearing on PAS data at any point over the follow-up. Prevalence of any HSC sight test and outcomes were determined as the proportion of the population recording an eye-test or ophthalmic health related outcome. Logistic regression, reporting odds ratios (OR) and 95% confidence intervals, firstly examined crude associations between SMI and the explanatory variables for each of the ophthalmic outcomes. Potential confounding of the association of SMI with each outcome was subsequently examined by including SMI with each additional explanatory variable in turn. Four fully adjusted models examined whether SMI was independently associated with any of the outcomes. In a further series of models, the interaction between SMI and age-group was examined. Likelihood ratio tests assessed effect modification at the 5% level of significance, by comparing the goodness of fit of models which included an SMI x age-group interaction term with null models. All analyses were produced using Stata version 14 [17].

## Results

### Sociodemographic characteristics of sample

Table 1 provides an overview of the hospital population characteristics as well as the prevalence of SMI, sight tests and ophthalmic-related health outcomes. Overall, 1.2% (N = 9,656) of the hospital population had an SMI diagnosis recorded between January 2013 and November 2019. Of those with SMI: 46.7% were male and 53.3% female (compared with 43.4% and 56.6% respectively in the non-SMI group); and they presented with a slightly older age profile when compared with the non-SMI group, especially between the ages of 35–64 years. SMI-diagnosed patients were less likely to be married/separated than the non-SMI group (25.5% and 44.0% respectively); and more likely to be single (39.8% and 20.2% respectively). SMI-diagnosed patients were more likely to live in urban rather than rural settings (27.9% and 21.3% respectively), while in the non-SMI group this was reversed (20.4% and 32.5% respectively). Finally, while the non-SMI patient group area-level deprivation shows an even distribution across all deciles (as expected with this population); with the SMI-diagnosed group however, the distribution of SMI increases in a graded way from 6.5% to 16.6% from least to most deprived respectively.

**Table 1:** Sociodemographic characteristics and ophthalmic treatment outcomes for both the SMI diagnosed and non-SMI diagnosed hospital population. The ophthalmic results are based on outcomes recorded between January 2015 and November 2019.

		non-SMI	SMI
		diagnosed	diagnosed
		patients	patients
		% (n)	% (n)
all (n = 798,564)		98.8 (788,908)	1.2 (9,656)
sex (n = 798,557)	male	43.4 (342,295)	46.7 (4,511)
	female	56.6 (446,608)	53.3 (5,143)
age group	15–24	8.9 (69,973)	8.8 (850)
	25–34	16.3 (128,373)	13.9 (1,345)
	35–44	14.7 (115,990)	15.9 (1,532)
	45–54	17.0 (133,773)	19.3 (1,860)
	55–64	15.7 (124,128)	16.5 (1,595)
	65–74	14.5 (114,140)	14.4 (1,388)
	75+	13.0 (102,531)	11.3 (1,086)
marital status	married/separated	44.0 (346,911)	25.5 (2,461)
	single	20.2 (159,407)	39.8 (3,839)
	divorced	2.2 (17,662)	4.9 (469)
	widowed	6.0 (47,488)	5.7 (552)
	not known	27.6 (217,440)	24.2 (2,335)
locale of residence (n = 798,437)	urban	20.4 (160,943)	27.9 (2,692)
	intermediate	47.1 (371,600)	50.8 (4,905)
	rural	32.5 (256,241)	21.3 (2,056)
deprivation decile (n = 798,437)	1 (most deprived)	9.9 (78,409)	16.6 (1,601)
	2	10.3 (81,078)	13.9 (1,346)
	3	10.2 (80,459)	11.5 (1,109)
	4	10.8 (85,065)	10.7 (1,036)
	5	10.8 (85,082)	10.2 (988)
	6	9.9 (77,966)	8.6 (825)
	7	10.2 (80,591)	8.7 (836)
	8	9.9 (78,047)	7.2 (692)
	9	9.0 (71,154)	6.2 (597)
	10 (least deprived)	9.0 (70,933)	6.5 (623)
any sight test	no	48.8 (384,820)	36.4 (3,528)
	yes	51.2 (404,088)	63.5 (6,128)
glaucoma ever noted	no	99.1 (781,475)	99.4 (9,597)
	yes	0.9 (7,433)	0.6 (59)
diabetes ever noted	no	95.0 (749,728)	93.5 (9,027)
	yes	5.0 (39,180)	6.5 (629)
any registered blindness	no	99.9 (788,000)	99.8 (9,638)
	yes	0.1 (908)	0.2 (18)

The 388,348 hospital patients who did not have an eye-test or did not appear on the ophthalmic register over this period were assumed not to have received dental treatment and were coded as zero on all dental outcomes.

Almost two-thirds (63.5%) of the SMI patients had an HSC eye-test over the follow-up period (compared with 51.2% non-SMI patients). The proportions recording specific ophthalmic-related health outcomes were as follows: glaucoma noted (0.6% v 0.9% in SMI patients and non-SMI patients respectively); diabetes (6.5% v 5.0% respectively); and registered blindness (0.2% v 0.1% respectively). Chi-squared tests comparing these rates confirm that SMI patients were more likely than non-SMI patients to have had a sight test (p<0.001), diabetes (p<0.001), and blindness (p = 0.041). However, rates of glaucoma were significantly lower among the SMI group (p = 0.001).

### Association of SMI with ophthalmic service use and related health outcomes

In univariable analysis (Table 2): compared to those recording no SMI, persons with SMI had a higher likelihood of any *sight test* (OR = 1.65: 95%CI = 1.59, 1.72), *diabetes* (OR = 1.33: 1.23, 1.45) or *registered blindness* (OR = 1.62: 1.02, 2.59), but a lower likelihood of *glaucoma* (OR = 0.65: 0.50, 0.84). Females were more likely than males to have had an eye-test and blindness, but less likely to record glaucoma or diabetes. Except for registered blindness, ageing was associated with generally uniformly increasing gradients for all outcomes. For eye-testing and diabetes, ORs increased until aged 65–74 years before reducing among those aged seventy-five years or older. Compared to persons married/separated: with the exception of blindness, single (never married) people recorded lower likelihoods for the other ophthalmic-related outcomes. Those previously married (divorced or widowed) were more likely to have had an eye-test and be recorded as blind. Glaucoma was more frequent amongst widowed people, as was diabetes in the divorced group. Rural dwellers were less likely to have had an eye-test (OR = 0.92: 0.91, 0.94), and had elevated risks for the other outcomes, except blindness (OR = 0.74: 0.61, 0.90). The likelihood of an eye-test and blindness generally decreased with lower levels of deprivation, while glaucoma was higher among individuals living in less deprived areas.

**Table 2:** Socio-demographic characteristics associated with selected ophthalmic health outcomes in the Northern Ireland hospital population. Data represents Odds Ratios (and 95% confidence intervals) from univariable logistic regression analyses.

	any eye-test	any glaucoma	any diabetes	any blindness
	OR (95% CI)	OR (95% CI)	OR (95% CI)	OR (95% CI)
SMI (reference = no)	1.00	1.00	1.00	1.00
yes	1.65 (1.59, 1.72)***	0.65 (0.50, 0.84)***	1.33 (1.23, 1.45)***	1.62 (1.02, 2.59)
sex (reference = male)	1.00	1.00	1.00	1.00
female	1.17 (1.16, 1.18)***	0.77 (0.73, 0.80)***	0.60 (0.59, 0.61)***	1.24 (1.08, 1.41)
age group (reference = 15–24)	1.00	1.00	1.00	1.00
25–34	0.87 (0.85, 0.89)***	2.02 (1.18, 3.46)	1.22 (1.11, 1.33)***	0.93 (0.68, 1.27)
35–44	1.47 (1.44, 1.50)***	12.73 (7.82, 20.70)***	3.36 (3.10, 3.65)***	1.13 (0.83, 1.53)
45–54	2.20 (2.15, 2.24)***	34.34 (21.27, 55.44)***	7.04 (6.51, 7.62)***	1.62 (1.23, 2.15)***
55–64	13.74 (13.44, 14.05)***	53.55 (33.21, 86.37)***	9.36 (8.65, 10.12)***	1.36 (1.01, 1.81)*
65–74	15.70 (15.34, 16.06)***	83.52 (51.82, 134.60)***	9.85 (9.10, 10.65)***	1.32 (0.98, 1.78)
75+	7.08 (6.93, 7.24)***	85.24 (52.88, 137.39)***	5.40 (4.98, 5.86)***	1.51 (1.13, 2.04)**
marital status (ref = married or separated)	1.00	1.00	1.00	1.00
single	0.55 (0.54, 0.56)***	0.33 (0.30, 0.36)***	0.50 (0.49, 0.52)***	1.12 (0.94, 1.33)
divorced	1.78 (1.72, 1.83)***	0.95 (0.81, 1.10)	1.22 (1.16, 1.30)***	2.03 (1.46, 2.83)***
widowed	2.08 (2.03, 2.12)***	1.47 (1.36, 1.59)***	1.00 (0.96, 1.04)	1.46 (1.13, 1.87)***
status not known	0.81 (0.81, 0.82)***	1.01 (0.96, 1.07)	0.78 (0.76, 0.80)***	1.07 (0.91, 1.26)
locale of residence (reference = urban)	1.00	1.00	1.00	1.00
intermediate	1.02 (1.00, 1.03)	1.25 (1.17, 1.33)***	1.20 (1.16, 1.23)***	1.22 (1.03, 1.44)
rural	0.92 (0.91, 0.94)***	1.25 (1.17, 1.34)***	1.10 (1.07, 1.13)***	0.74 (0.61, 0.90)**
deprivation decile (ref = most deprived)	1.00	1.00	1.00	1.00
decile 2	0.94 (0.92, 0.96)***	1.31 (1.16, 1.47)***	1.09 (1.04, 1.14)***	0.92 (0.73, 1.17)
3	0.90 (0.88, 0.92)***	1.54 (1.37, 1.73)***	1.08 (1.03, 1.13)***	0.95 (0.75, 1.21)
4	0.87 (0.86, 0.89)***	1.37 (1.22, 1.54)***	1.03 (0.99, 1.08)	0.85 (0.67, 1.08)
5	0.86 (0.84, 0.87)***	1.65 (1.48, 1.85)***	1.08 (1.03, 1.12)***	0.45 (0.34, 0.60)***
6	0.86 (0.84, 0.87)***	1.54 (1.37, 1.72)***	1.03 (0.99, 1.09)	0.46 (0.34, 0.62)***
7	0.81 (0.79, 0.83)***	1.70 (1.52, 1.90)***	1.07 (1.03, 1.12)**	0.62 (0.47, 0.80)***
8	0.82 (0.81, 0.84)***	1.65 (1.47, 1.85)***	1.01 (0.97, 1.06)	0.42 (0.31, 0.57)***
decile 9	0.83 (0.82, 0.85)***	1.90 (1.70, 2.13)***	1.02 (0.98, 1.07)	0.55 (0.41, 0.73)***
10 (least deprived)	0.85 (0.84, 0.87)***	2.04 (1.83, 2.28)***	0.90 (0.86, 0.95)***	0.46 (0.34, 0.63)***

***: p< = 0.001;

**: p< = 0.005;

*: p< = 0.05

Following full adjustment for available socio-demographic characteristics (Table 3), eye-testing and diabetes remained more likely among the SMI group (OR = 1.71: 95%CI = 1.63, 1.79 and OR = 1.29: 1.19, 1.40 respectively); while glaucoma remained less likely (OR = 0.69: 0.53, 0.90). There was no association recorded between SMI and blindness in the fully adjusted model. Comparing the unadjusted and adjusted ORs associated with SMI (row one of Tables 2 and 3): with the exception of *blindness*, full adjustment does not materially alter the likelihoods associated with each of these ophthalmic outcomes (their respective confidence intervals remain overlapping in both models)–suggesting that these outcomes are relatively independent of circumstance; however, with *blindness* the addition of the socio-demographic indicators reduces the associated likelihood and reduces it to non-significance.

**Table 3:** Fully adjusted logistic regression models showing association of SMI with the selected ophthalmic health outcomes (N = 798,430).

	any eye-test	any glaucoma	any diabetes	any blindness
	OR (95% CI)	OR (95% CI)	OR (95% CI)	OR (95% CI)
SMI (reference = no)	1.00	1.00	1.00	1.00
yes	1.71 (1.63, 1.79)***	0.69 (0.53, 0.90)**	1.29 (1.19, 1.40)***	1.39 (0.87, 2.21)
sex (reference = male)	1.00	1.00	1.00	1.00
female	1.61 (1.59, 1.62)***	0.88 (0.84, 0.92)***	0.67 (0.66, 0.69)***	1.27 (1.11, 1.45)***
age group (reference = 15–24)	1.00	1.00	1.00	1.00
25–34	0.94 (0.92, 0.96)***	1.96 (1.14, 3.34)*	1.22 (1.11, 1.33)***	0.98 (0.72, 1.34)
35–44	1.80 (1.76,1.84)***	11.69 (7.18, 19.04)***	3.23 (2.96, 3.51)***	1.28 (0.94, 1.75)
45–54	2.85 (2.79, 2.91)***	30.99 (19.16, 50.12)***	6.66 (6.14, 7.22)***	1.88 (1.40, 2.53)***
55–64	19.42 (18.95, 19.89)***	47.76 (29.56, 77.19)***	8.73 (8.05, 9.47)***	1.60 (1.18, 2.19)**
65–74	22.69 (22.12, 23.27)***	75.13 (46.52, 121.34)***	9.22 (8.50, 10.00)***	1.57 (1.15, 2.16)**
75+	10.10 (9.85,10.35)***	80.42 (49.78, 129.91)***	5.24 (4.82, 5.70)***	1.73 (1.25, 2.39)***
marital status (reference = married/separated)	1.00	1.00	1.00	1.001.24 (1.02,
single	1.42 (1.40, 1.44)***	0.85 (0.78, 0.94)***	0.94 (0.91, 0.97)***	1.50)*
divorced	1.37 (1.32, 1.42)***	0.91 (0.78, 1.06)	1.04 (0.98, 1.11)	1.60 (1.15, 2.23)**
widowed	0.80, 0.84)***	0.83 (0.76, 0.91)***	0.98 (0.94, 1.03)	1.20 (0.91, 1.58)
unknown	1.00 (0.99, 1.01)	1.15 (1.09, 1.21)***	0.84 (0.82, 0.86)***	1.10 (0.94, 1.30)
locale of residence (reference = urban)	1.00	1.00	1.00	1.00
intermediate	1.04 (1.03, 1.06)***	1.09 (1.02, 1.17)*	1.17 (1.14, 1.21)***	1.45 (1.21, 1.74)***
rural	(0.91, 0.94)***	1.10 (1.02, 1.18)*	1.03 (0.99, 1.06)	0.95 (0.77, 1.19)
deprivation decile (reference = most deprived)	1.00	1.00	1.00	1.00
decile 2	0.86 (0.84, 0.88)***	1.16 (1.03, 1.31)	1.01 (0.96, 1.06)	0.86 (0.67, 1.10)
3	0.77 (0.75, 0.79)***	1.29 (1.14, 1.45)***	0.95 (0.91, 1.00)	0.86 (0.67, 1.11)
4	0.73 (0.72, 0.75)***	1.13 (1.00, 1.28)	0.91 (0.87, 0.96)***	0.81 (0.63, 1.05)
5	0.71 (0.70, 0.73)***	1.35 (1.20, 1.52)***	0.95 (0.91, 1.00)	0.44 (0.32, 0.60)***
6	0.71 (0.69, 0.73)***	1.24 (1.10, 1.40)***	0.91 (0.87, 0.96)***	0.45 (0.33, 0.62)***
7	0.65 (0.64, 0.67)***	1.36 (1.21, 1.54)***	0.94 (0.89, 0.96)	0.57 (0.43, 0.76)***
8	0.63 (0.62, 0.65)***	1.28 (1.13, 1.44)***	0.82, 0.90)	0.40 (0.29, 0.55)***
decile 9	0.60 0.59, 0.61)***	1.43 (1.27, 1.61)***	0.83 (0.80, 0.88)	0.48 (0.35, 0.65)***
10 (least deprived)	0.55 (0.54, 0.57)***	1.47 (1.31, 1.65)***	0.72 (0.69, 0.76)	0.40 (0.29, 0.54)***

***: p< = 0.001;

**: p< = 0.005;

*: p< = 0.05

### Interaction of SMI and age-group

Effect modification by age-group of the association between SMI, and (separately) any eye-test and diabetes was explored through the addition of an interaction term in fully adjusted models (Table 4). For glaucoma and blindness, because of sparsely populated cells relating to the interaction terms, it was not possible to complete this sub-analysis. Likelihood ratio (LR) tests compared the goodness of fit of each null model with a model including the interaction term (*SMI* x age-group) for both outcomes. There was evidence of effect modification by age-group: for models relating to any eye-testing and diabetes the LR chi^2^ values were 1025.22 and 89.77 respectively, with p<0.001 for each comparison. Individuals with SMI in older age-groups were less likely to have had an eye-test, in contrast to the general trend of increasing odds among older aged individuals in the respective null model. While likelihood of diabetes generally increased with age (as shown in the null model in Table 3), interaction analysis suggests that this trend was not mirrored among individuals with SMI.

**Table 4:** Logistic regression models showing SMI and age-group interactions, and their association with selected ophthalmic health outcomes. All models are fully adjusted for sex, marital status, locale of residence and area level deprivation.

	any eye-test	any diabetes
	OR (95% CI)	OR (95% CI)
SMI by age-group		
(ref SMI x 15–24)	1.00	1.00
SMI x 25–34	1.12 (0.94, 1.33)	1.90 (0.93, 3.87)
SMI x 35–44	1.07 (0.90, 1.27)	1.84 (0.95, 3.57)
SMI x 45–54	0.89 (0.74, 1.05)	1.51 (0.79, 2.88)
SMI x 55–64	0.21 (0.17, 0.24)***	1.04 (0.54, 2.00)
SMI x 65–74	0.19 (0.16, 0.23)***	0.63 (0.32, 1.22)
SMI x 75 plus	0.30 (0.25, 0.36)***	0.64 (0.32, 1.29)

***: p< = 0.001;

**: p< = 0.005;

*: p< = 0.05

## Discussion

This study adds to the growing evidence on health disparities for people with severe and enduring mental illness. It also points to some complexity in the relationship between SMI and ophthalmic health-related outcomes. In contrast to wider evidence of lower levels of service use among SMI patients [18], we found higher levels of eye-tests among SMI patients after controlling for age and sex and other socio-demographic characteristics. Consistent with earlier research [19], we also found higher likelihood of diabetes mellitus noted in ophthalmic healthcare settings. However, glaucoma was *less likely* among SMI patients, despite higher rates of eye-testing and some previous evidence of an association between poor mental health and glaucoma [11, 12]. Lower glaucoma diagnosis among SMI patients may reflect their younger age profile. Glaucoma can affect people at all ages but is most common in adults aged seventy or more [20]. Nonetheless, our findings may point to undetected glaucoma among patients with SMI. While our analysis indicates higher levels of eye examinations among SMI patients *per se*, interaction analysis based on age-group shows that older individuals with SMI were *less likely* to have had an eye-test. These findings mirror earlier results from recent research on SMI and dental health outcomes [21], and highlight likely unmet need among older patients with SMI. A growing body of evidence also points to the contribution of sub-optimal physical healthcare provision to poorer physical outcomes for people with SMI [2, 5, 22]. Analysis presented in this study, together with earlier findings in relation to dental health outcomes [21], highlights potential inequalities in provision of physical healthcare, particularly amongst *older people with SMI*, which merits further investigation.

The higher rates of diabetes found in SMI patients in ophthalmic healthcare settings underlines the need for more assertive and routine diabetes screening in this population, ensuring early detection and intervention. Public Health England also reported higher rates of diabetes among SMI patients, as well as elevated rates of cardiovascular, respiratory, and other life-limiting conditions [13]. Such conditions are known to be influenced by lifestyle factors such as poor diet, lack of exercise and substance misuse, which are pertinent challenges among individuals with SMI [23]. We are unable to state if the higher rates of blindness are related to diabetes but there is an obvious possibility that this is the case.

Importantly, our study also identifies key socio-demographic characteristics associated with ophthalmic service use and related health outcomes: for example, it points to gender differences, with females more likely to have had an eye-test and recorded blindness, but less likely to have glaucoma or diabetes recorded. Higher risk of blindness among females, due to longevity has been consistently reported [24]; while the association of gender with glaucoma and diabetes is more complex, and dependent on condition sub-types as well as a range of modifiable and non-modifiable risk factors [25, 26]. Our findings also highlight urban-rural inequity, with lower levels of eye-testing among rural dwellers, but higher risk of adverse ophthalmic-related health outcomes. This finding, which merits further research into underdiagnosed ophthalmic health problems in rural communities, fits in a wider context of rural/urban disparities in access to healthcare services [27]. We also found higher levels of eye examinations among those who were previously married, with higher levels of glaucoma among the widowed group and higher levels of diabetes in the divorced group. While higher rates of glaucoma among widows is likely to reflect the higher prevalence of the condition among older people, research points to the association of environmentally modifiable risk factors such as lifestyle, exercise, and nutrition [28, 29], which may play an important role for this sub-group.

### Strengths and limitations

There is scant research examining the association between SMI and ophthalmic health. This is the first population-based administrative data study from within the UK to examine ophthalmic outcomes among people with SMI, with the findings highlighted here contributing to the developing evidence-base through the linkage of large clinically relevant datasets, using internationally recognized diagnoses to define the population at risk, and ophthalmic related health outcomes identified in clinical settings. However, we acknowledge that in our study some groups of hospital patients may be over-represented—for example, those in the youngest and oldest age groups. Moreover, private patients are excluded in these data, with only those on benefits or lower levels of income accepted as NHS patients; this is likely to lessen the true disparities in relation to general population ophthalmic data. Furthermore, at each sight-test, only one eligibility criterion is recorded on the electronic record and therefore patients with both glaucoma and diabetes (for example), will record only one of these indicators at each attendance. Although we examined health outcomes over a five-year period, the cross-sectional nature of the study limits any causal inferences. Moreover, as with most administrative data studies, we had limited explanatory covariates to further our understanding of personal characteristics and contextual factors.
